# Deep Learning for Predicting Biomolecular Binding Sites of Proteins

**DOI:** 10.34133/research.0615

**Published:** 2025-02-24

**Authors:** Minjie Mou, Zhichao Zhang, Ziqi Pan, Feng Zhu

**Affiliations:** College of Pharmaceutical Sciences, The Second Affiliated Hospital, Zhejiang University School of Medicine, National Key Laboratory of Advanced Drug Delivery and Release Systems, Zhejiang University, Hangzhou 310058, China.

## Abstract

The rapid evolution of deep learning has markedly enhanced protein–biomolecule binding site prediction, offering insights essential for drug discovery, mutation analysis, and molecular biology. Advancements in both sequence-based and structure-based methods demonstrate their distinct strengths and limitations. Sequence-based approaches offer efficiency and adaptability, while structure-based techniques provide spatial precision but require high-quality structural data. Emerging trends in hybrid models that combine multimodal data, such as integrating sequence and structural information, along with innovations in geometric deep learning, present promising directions for improving prediction accuracy. This perspective summarizes challenges such as computational demands and dynamic modeling and proposes strategies for future research. The ultimate goal is the development of computationally efficient and flexible models capable of capturing the complexity of real-world biomolecular interactions, thereby broadening the scope and applicability of binding site predictions across a wide range of biomedical contexts.

## Introduction

Recent advancements in sequence-based and structure-based deep learning approaches have markedly propelled the field of binding site prediction, offering profound insights into protein interactions and molecular mechanisms. These developments have accelerated key applications in target identification, mutation analysis, and drug design [1,2]. Furthermore, these advances highlight emerging trends, identify critical challenges, and outline forward-thinking directions for future research and practical implementation.

## Advances in Binding Site Prediction

Binding site prediction methods are generally classified into 2 categories: sequence-based and structure-based approaches, each with its own advantages and limitations (Table). Sequence-based methods leverage amino acid sequences and evolutionary information, primarily focusing on linear sequence features [3–5]. Conversely, structure-based approaches rely on 3-dimensional protein structures to capture spatial arrangements crucial to binding interactions [6]. High-precision models, such as those in the AlphaFold series, provide the structural data needed for accurate binding site identification [7]. The integration of sequence and structural data has become a key trend for increasing prediction accuracy [8,9].

**Table.** Comparison of sequence-based and structure-based binding site prediction methods. The methodologies for predicting protein–biomolecule binding sites are categorized into sequence-based and structure-based approaches. The comparison includes representative models, highlights key features, and evaluates advantages and limitations.

Type	Subcategory	Models	Key features	Advantages	Limitations
Sequence-based methods	Transformer-based methods	EnsemPPIS, PepBCL	Leverages attention mechanisms and pretrained models to capture long-range sequence dependencies	Excels at capturing complex sequence relationships; highly adaptable to various tasks	High computational cost for long sequences
	CNN-based methods	DeepDISOBind, DELPHI	Uses convolutional layers to identify local sequence features, ideal for detecting motifs	Efficiently extracts local features; suitable for small datasets with minimal computing costs	Limited in capturing global sequence information
	Other sequence-based methods	ESM-DBP, SAResNet	Applies diverse neural networks (e.g., RNN, ResNet) for flexible and customizable sequence analysis	Customizable for specific tasks; easily integrates with biological features and databases	Relies on quality input features and sensitive to noisy or limited data
Structure-based methods	Geometric deep learning methods	DeepGlycanSite, GeoBind	Constructs 3-dimension protein representations using point clouds or surface graphs	Handles complex surface shapes effectively for protein binding site analysis	Relies on data diversity; costly for large inputs
	GNN-based methods	EGRET, MEG-PPIS	Models proteins as residue graphs, capturing spatial and topological features effectively	Excels at integrating local and global residue interactions with detailed constraints	High complexity; sensitive to graph generation quality
	Surface property-based methods	MaSIF, PeSTo	Analyzes surface properties like electrostatics, and hydrophobicity via point clouds or meshes	Effectively and precisely identifies binding sites based on surface properties	May not capture internal structural information

Geometric deep learning provides flexible ways to model protein structures by leveraging local and global geometric relationships. Point cloud models capture detailed spatial features of complex binding interfaces [6,10,11]. Surface property-based methods focus on overall surface features, such as hydrophobic patches or charge distributions, critical for protein interactions [12,13]. Graph neural networks (GNNs) encode proteins as graphs, incorporating physicochemical constraints and spatial relationships to improve binding predictions [11,14,15].

Transformer-based approaches capture long-range dependencies in sequences using attention mechanisms and pretrained models, making them effective for complex sequence relationships. However, they are computationally intensive, especially with long sequences, as seen in methods like PepBCL. Convolutional neural network (CNN)-based approaches focus on local sequence patterns using convolutional layers, which is efficient for motif detection in smaller datasets. However, they struggle to capture global sequence dependencies, as demonstrated by methods like DELPHI.

Multi-task frameworks, like DeepDISOBind, capture shared features across different interaction types, such as RNA, DNA, and proteins [3,16]. Additionally, ensemble learning frameworks enhance model robustness by combining diverse neural network architectures, as seen in EnsemPPIS, which integrates Transformer and gated CNNs to effectively capture both global and local interaction features within protein sequences [17]. Recently, advanced protein language models (PLMs) have also been applied to binding site prediction, such as ESM-DBP, which can substantially enhance prediction accuracy [18,19].

## Challenges and Prospects in Enhancing Binding Site Prediction

Both sequence-based and structure-based methods, despite substantial advances, have certain limitations. Structure-based methods, while highly accurate, rely heavily on high-quality structural data, often obtained from experiments or advanced prediction tools like AlphaFold [7,20]. However, even AlphaFold’s high-precision predictions may not fully capture protein dynamics in complex biological contexts, where conformational changes and environmental factors influence binding. Furthermore, structure-based methods are inherently limited in addressing protein mutations that modify the protein’s 3-dimensional configuration, underscoring the need for flexible models that can incorporate structural dynamics and account for binding site alterations. For instance, many biological processes, including enzyme catalysis and molecular signaling, require the consideration of protein flexibility and transient conformational states, which cannot always be captured by static structural models. These factors highlight the ongoing challenge of integrating structural dynamics into binding site prediction models, which could better reflect the complexity of biological systems.

In contrast, sequence-based methods offer computational efficiency and adaptability, making them valuable in scenarios where structural data are unavailable. These models leverage amino acid sequences and evolutionary conservation to identify binding residues but often struggle to capture spatial features crucial for precise predictions [21]. Although these methods excel in efficiency, they fall short in capturing the spatial context of protein interactions, which limits their prediction accuracy. To mitigate this limitation, incorporating spatial constraints such as predicted residue–residue interactions or sequence-based structural motifs could provide a more nuanced understanding of binding sites without relying on structural data. This flexibility enables dynamic predictions, particularly when proteins undergo conformational changes, positioning sequence-based approaches as valuable tools for studying mutation impacts. Incorporating such spatial and dynamic elements would not only improve prediction accuracy but also enhance the model’s robustness across diverse biological conditions, expanding the potential applications of sequence-based methods. Given their simplicity, efficiency, and adaptability, sequence-based methods warrant further research, particularly as they evolve to integrate spatial and dynamic features.

In the future, dynamically integrating sequence and structural data holds potential for advancing binding site prediction. Hybrid models that combine sequence specificity with structural context can more effectively capture a broad spectrum of biomolecular interactions [10,15]. Multi-task learning and ensemble frameworks, which leverage shared features across tasks and combine the strengths of individual models, offer promising strategies to achieve this integration. In particular, ensemble frameworks enhance adaptability and robustness across diverse biomolecular interactions [17,22].

In fact, the successful integration of multiple data modalities has already demonstrated substantial improvements in related fields. A notable example is SurfDock [23], which combines sequence, structural, and physicochemical information to enhance protein–ligand binding pose and affinity predictions. By leveraging this multi-faceted approach, SurfDock has achieved a remarkable 20% improvement in predicting binding affinities over traditional single-modal methods. This highlights the promising potential of multi-modal integration, providing compelling evidence that such an approach could substantially advance the accuracy and reliability of protein–biomolecule binding site predictions.

Furthermore, while some models already integrate basic physicochemical properties such as charge distribution and hydropathy, advancements in molecular science can facilitate the inclusion of more complex molecular properties that offer potential for improving prediction accuracy. For example, with the development of advanced experimental techniques like molecular dynamics simulations and cryo-electron microscopy, researchers are now able to capture real-time atomic-level interactions and transient molecular states that are critical for accurate protein–biomolecule interaction predictions. These properties, such as the flexibility of binding interfaces or the detailed molecular forces at play, are often overlooked in traditional static models. By further incorporating these dynamic factors, as shown in the Figure, prediction models could provide a more refined and realistic depiction of protein–biomolecule interactions. This is especially important in capturing molecular flexibility and transient binding events, which play key roles in biological processes like enzyme catalysis, molecular signaling, and protein folding. Moreover, integrating these complex properties can enhance model robustness by accounting for the inherent variability and dynamism of biological systems. As a result, these enhanced models could offer greater accuracy in predicting binding sites, thus pushing the boundaries of current prediction technologies [18,24,25].

**Figure.**
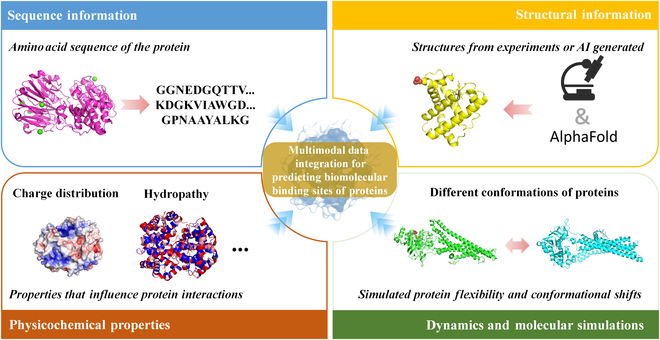
Multimodal data integration for predicting biomolecular binding sites of proteins. The integration of multimodal data can enhance the prediction of biomolecular binding sites of proteins. Sequence information, derived from amino acid sequences, provides critical insights into binding residues and evolutionary patterns. Structural information, obtained from experimental techniques or artificial intelligence (AI)-driven tools such as AlphaFold, offers spatial precision for identifying binding sites. Physicochemical properties, including charge distribution and hydropathy, influence protein interactions. Dynamics and molecular simulations capture protein flexibility and transient conformational states, enabling the study of dynamic molecular interactions. Collectively, these data modalities provide a comprehensive framework for improving the accuracy of binding site predictions in complex biological systems.

Achieving computational efficiency remains crucial given the increasing complexity of deep learning models. Developing lightweight models that maintain high accuracy while reducing computational demands is essential, particularly in data-limited or dynamic prediction settings [26]. Sequence-based methods are particularly promising in this regard, as they are inherently less computationally intensive. Future work may focus on streamlined architectures capable of capturing essential spatial and evolutionary patterns without sacrificing accuracy. Incorporating efficient neural architectures like Transformers and deep reinforcement learning (RL) could speed up training and improve generalization across diverse protein sequences, addressing model complexity. Additionally, future research might explore reducing dependency on multiple sequence alignment (MSA) or developing MSA-independent alternatives. Such innovations could lower computational costs and expedite data processing, thereby enhancing model applicability across a broader range of prediction contexts.

## Conclusion

Advancements in deep learning have profoundly enhanced protein–biomolecule binding site prediction. Hybrid models that seamlessly integrate sequence and structural data promise to substantially enhance predictive accuracy, mitigating the limitations inherent in individual approaches. Furthermore, the strategic integration of lightweight architectures and multimodal data will optimize computational efficiency. Continued progress in these areas will broaden the impact of binding site prediction, driving transformative advances in drug target identification, mutation analysis, and therapeutic development.

## References

[1] Senior AW, Evans R, Jumper J, Kirkpatrick J, Sifre L, Green T, Qin C, Zidek A, Nelson AWR, Bridgland A, et al. Improved protein structure prediction using potentials from deep learning. Nature. 2020;577(7792):706–710. 31942072 10.1038/s41586-019-1923-7

[2] Gainza P, Wehrle S, Van Hall-Beauvais A, Marchand A, Scheck A, Harteveld Z, Buckley S, Ni D, Tan S, Sverrisson F, et al. De novo design of protein interactions with learned surface fingerprints. Nature. 2023;617(7959):176–184. 37100904 10.1038/s41586-023-05993-x PMC10131520

[3] Zhang F, Zhao B, Shi W, Li M, Kurgan L. DeepDISOBind: Accurate prediction of RNA-, DNA- and protein-binding intrinsically disordered residues with deep multi-task learning. Brief Bioinform. 2022;23(1):bbab521. 34905768 10.1093/bib/bbab521

[4] Shen LC, Liu Y, Song JN, Yu DJ. SAResNet: Self-attention residual network for predicting DNA-protein binding. Brief Bioinform. 2021;22(5):bbab101. 33837387 10.1093/bib/bbab101 PMC8579196

[5] Huang JX, Li WK, Xiao B, Zhao CQ, Zheng HC, Li YR, Wang J. PepCA: Unveiling protein-peptide interaction sites with a multi-input neural network model. Iscience. 2024;27(10): Article 110850. 39391726 10.1016/j.isci.2024.110850 PMC11465048

[6] He X, Zhao L, Tian Y, Li R, Chu Q, Gu Z, Zheng M, Wang Y, Li S, Jiang H, et al. Highly accurate carbohydrate-binding site prediction with DeepGlycanSite. Nat Commun. 2024;15(1):5163. 38886381 10.1038/s41467-024-49516-2 PMC11183243

[7] Jumper J, Evans R, Pritzel A, Green T, Figurnov M, Ronneberger O, Tunyasuvunakool K, Bates R, Zidek A, Potapenko A, et al. Highly accurate protein structure prediction with AlphaFold. Nature. 2021;596(7873):583–589. 34265844 10.1038/s41586-021-03819-2 PMC8371605

[8] Yuan Q, Chen S, Rao J, Zheng S, Zhao H, Yang Y. AlphaFold2-aware protein-DNA binding site prediction using graph transformer. Brief Bioinform. 2022;23(2):bbab564. 35039821 10.1093/bib/bbab564

[9] Shafiee S, Fathi A, Taherzadeh G. DP-site: A dual deep learning-based method for protein-peptide interaction site prediction. Methods. 2024;229:17–29. 38871095 10.1016/j.ymeth.2024.06.001

[10] Xia Y, Xia CQ, Pan X, Shen HB. GraphBind: Protein structural context embedded rules learned by hierarchical graph neural networks for recognizing nucleic-acid-binding residues. Nucleic Acids Res. 2021;49(9): Article e51. 33577689 10.1093/nar/gkab044 PMC8136796

[11] Li P, Liu ZP. GeoBind: Segmentation of nucleic acid binding interface on protein surface with geometric deep learning. Nucleic Acids Res. 2023;51(10): Article e60. 37070217 10.1093/nar/gkad288 PMC10250245

[12] Gainza P, Sverrisson F, Monti F, Rodola E, Boscaini D, Bronstein MM, Correia BE. Deciphering interaction fingerprints from protein molecular surfaces using geometric deep learning. Nat Methods. 2020;17(2):184–192. 31819266 10.1038/s41592-019-0666-6

[13] Krapp LF, Abriata LA, Cortes Rodriguez F, Dal Peraro M. PeSTo: Parameter-free geometric deep learning for accurate prediction of protein binding interfaces. Nat Commun. 2023;14(1):2175. 37072397 10.1038/s41467-023-37701-8 PMC10113261

[14] Mahbub S, Bayzid MS. EGRET: Edge aggregated graph attention networks and transfer learning improve protein-protein interaction site prediction. Brief Bioinform. 2022;23(2):bbab578. 35106547 10.1093/bib/bbab578

[15] Ding H, Li X, Han P, Tian X, Jing F, Wang S, Song T, Fu H, Kang N. MEG-PPIS: A fast protein-protein interaction site prediction method based on multi-scale graph information and equivariant graph neural network. Bioinformatics. 2024;40(5):btae269. 38640481 10.1093/bioinformatics/btae269 PMC11252844

[16] Wang N, Yan K, Zhang J, Liu B. iDRNA-ITF: Identifying DNA- and RNA-binding residues in proteins based on induction and transfer framework. Brief Bioinform. 2022;23(4):bbac236. 35709747 10.1093/bib/bbac236

[17] Mou M, Pan Z, Zhou Z, Zheng L, Zhang H, Shi S, Li F, Sun X, Zhu F. A transformer-based ensemble framework for the prediction of protein-protein interaction sites. Research. 2023;6:0240. 37771850 10.34133/research.0240 PMC10528219

[18] Zeng W, Dou Y, Pan L, Xu L, Peng S. Improving prediction performance of general protein language model by domain-adaptive pretraining on DNA-binding protein. Nat Commun. 2024;15(1):7838. 39244557 10.1038/s41467-024-52293-7 PMC11380688

[19] Liu YF, Tian BX. Protein-DNA binding sites prediction based on pre-trained protein language model and contrastive learning. Brief Bioinform. 2024;25(1):bbad488. 10.1093/bib/bbad488 PMC10782905 38171929

[20] Alam R, Mahbub S, Bayzid MS. Pair-EGRET: Enhancing the prediction of protein-protein interaction sites through graph attention networks and protein language models. Bioinformatics. 2024;40(10):btae588. 39360982 10.1093/bioinformatics/btae588 PMC11495673

[21] Wang R, Jin J, Zou Q, Nakai K, Wei L. Predicting protein-peptide binding residues via interpretable deep learning. Bioinformatics. 2022;38(13):3351–3360. 35604077 10.1093/bioinformatics/btac352

[22] Zeng M, Zhang F, Wu FX, Li Y, Wang J, Li M. Protein-protein interaction site prediction through combining local and global features with deep neural networks. Bioinformatics. 2020;36(4):1114–1120. 31593229 10.1093/bioinformatics/btz699

[23] Cao DH, Chen MG, Zhang RZ, Wang ZK, Huang ML, Yu J, Jiang XY, Fan ZH, Zhang W, Zhou H, et al. SurfDock is a surface-informed diffusion generative model for reliable and accurate protein-ligand complex prediction. Nat Methods. 2024. 10.1038/s41592-024-02516-y 39604569

[24] Yin S, Mi X, Shukla D. Leveraging machine learning models for peptide-protein interaction prediction. RSC Chem Biol. 2024;5(5):401–417. 38725911 10.1039/d3cb00208j PMC11078210

[25] Li Y, Golding GB, Ilie L. DELPHI: Accurate deep ensemble model for protein interaction sites prediction. Bioinformatics. 2021;37(7):896–904. 32840562 10.1093/bioinformatics/btaa750

[26] Baranwal M, Magner A, Saldinger J, Turali-Emre ES, Elvati P, Kozarekar S, VanEpps JS, Kotov NA, Violi A, Hero AO. Struct2Graph: A graph attention network for structure based predictions of protein-protein interactions. BMC Bioinformatics. 2022;23(1):370. 36088285 10.1186/s12859-022-04910-9 PMC9464414

